# Utility of self-competency ratings during residency training in family medicine education-emerging countries: findings from Japan

**DOI:** 10.1186/s12930-016-0031-1

**Published:** 2017-01-10

**Authors:** Michael D. Fetters, Satoko Motohara, Lauren Ivey, Keiichiro Narumoto, Kiyoshi Sano, Masahiko Terada, Tsukasa Tsuda, Machiko Inoue

**Affiliations:** 1 Department of Family Medicine, University of Michigan, 1018 Fuller Street, Ann Arbor, MI 48104-1213 USA; 2 School of Medicine, Loma Linda University, Loma Linda, CA USA; 3 Department of Obstetrics/Gynecology and Family Medicine, Hamamatsu University, Hamamatsu, Japan; 4 Shizuoka Family Medicine Training Program, Shizuoka, Japan; 5 Department of Family Medicine, Tokushukai Medical Corporation, Shizuoka, Japan; 6 Department of Family and Community Medicine, Hamamatsu University, Hamamatsu, Japan

**Keywords:** Residency training, Evaluation, Competency, Developing countries, Japan, Family medicine, General medicine, Post-graduate education, Primary care

## Abstract

**Background:**

Family medicine education-emerging countries face challenges in demonstrating a new program’s ability to train residents in womb-to-tomb care and resident ability to provide such care competently. We illustrate the experience of a new Japanese family medicine program with resident self-competency assessments.

**Methods:**

In this longitudinal cross-sectional study, residents completed self-competency assessment surveys online during 2011–2015. Each year of training, residents self-ranked their competence using a 100-point visual analog scale for 142 conditions: acute (30 conditions), chronic (28 conditions) women’s health (eight conditions), and geriatrics/home (12 conditions) care; procedures (38 types); health promotion (21 conditions).

**Results:**

Twenty residents (11 women, 9 men) participated. Scores improved annually by training year from baseline to graduation; the mean composite score advanced from 31 to 65%. All subcategories showed improvement. Scores for care involving acute conditions rose from 49 to 75% (26% increase); emergency procedures, 46–65% (19% increase); chronic care, 33–73% (40% increase); women’s health, 16–59% (43% increase); procedural care, 26–56% (30% increase); geriatrics care-procedures, 8–65% (57% increase); health promotion, 21–63% (42% increase). Acute care, chronic care, and health promotion achieved the highest levels. Women’s health care, screenings, and geriatrics experienced the greatest increase. Health promotion gains occurred most dramatically in the final residency year.

**Conclusions:**

A resident self-competency assessment provides a simple and practical way to conduct an assessment of skills, to monitor skills over time, to use the data to inform residency program improvement, and to demonstrate the breadth of family medicine training to policymakers, and other stakeholders.

## Background

Organized general/family medicine (FM) as a recognized medical specialty has a decades’ long history in multiple countries, dating back to the 1950s in the United Kingdom [1], Canada [2] and Australia [3] and to 1969 in the United States [4]. Still, in many countries, FM is unrecognized or just emerging as a specialty [5–9]. In such places FM programs often lack resources for teaching and evaluating residents using the types of intensive one-on-one teaching procedures and complex competency or milestone evaluations that characterize FM education in more experienced countries.

FM programs may emerge because of policy changes that encourage or mandate their development. In this report, we refer to countries just beginning to develop systems or lacking official government-approved FM programs as *Family Medicine Education Emerging (FMEE)* countries. *Emerging* emphasizes that sociopolitical forces have reached a level where the determination across various constituencies to launch FM training is sufficient, relatively few faculty exist, and previous FM teaching experience is limited. FMEE academic programs face many barriers in launching a new discipline that impacts training at the student, resident, and faculty levels.

In Japan, a FMEE country, FM has not been recognized by the Japanese government as a medical specialty despite the efforts of advocates since the 1980s [10]. However, some recent crises have created a sociopolitical climate favoring FM development. Japan is a superaging society, and the rapid growth in the elderly population has highlighted significant problems: fragmentation of care; polypharmacy; need for after-hours care, including home and palliative care; and sky-rocketing health care costs.

In addition to the need for FM specialists [10] and status reports [11, 12], other literature emphasizes the potential role of FM in the community [13], academic medical centers [14], and especially to support women’s health [15]. Acknowledging Japan as a superaging society with fragmented medical care, the Japan Ministry of Health Labour and Welfare has articulated the clear need the need for general/FM physicians who can manage care efficiently, competently handle multiple medical problems, coordinate care, and address biopsychocial issues [16], and laid out plans for establishing general/FM as the 19th official specialty in Japan effective 2017 [17].

In light of new World Federation of Medical Education standards, the newly formed Japan Accreditation Council for Medical Education (JACME) will require medical schools to comply with new accreditation standards by 2023, including a newly required general medicine clinical rotation. Moreover, dedicated health tracks in medical schools focused on placing young physicians in health care shortage areas have seen limited success, despite lenient admission criteria, tuition waivers, and scholarships to individuals from health care shortage areas. These factors are forcing academicians to accept community and general medicine training as needed components of medical school training. The push from FM advocates and the crises confronting policymakers and academic leaders have provided the sociopolitical environment needed. In 2013, the Japanese government announced, as part of its restructuring of the specialist certification process, that a generalist specialty physician, called *sogou shinryoui*, will be recognized as the 19th Japanese medical specialty, starting in 2017 [18].

Despite national policy interest and academic medical institutions’ motivation to maintain international accreditation by offering clinical teaching in family/general medicine in Japan, some policy makers, academicians and subspecialists have been reluctant to change the status quo. Without previous experience working with or training family physicians, there may be apprehension as to whether FM residents can be trained across multiple disciplines and acquire the breadth of knowledge and skills required to treat the full spectrum of common conditions. Apprehension like this, which is not unique to Japan, challenges FMEE countries to provide evidence relatively quickly, despite limited resources, about the impact of FM training and to demonstrate programs’ ability to produce residents with the skills needed to provide competent womb-to-tomb care.

In medical residency training, *competency* is considered “an observable ability of a health professional, integrating multiple components such as knowledge, skills, values, and attitudes [19]. As a part of their Outcome Project, in 1999 the U.S. Accreditation Council for Graduate Medical Education (ACGME) defined six core competencies expected of medical residents: patient care, medical knowledge, interpersonal and communication skills, professionalism, and practice-based medicine [20]. Resident training programs must demonstrate that residents are developing these competencies by providing educational experiences and valid and reliable tools to use as evaluation instruments [21]. Along with contextualizing the concept of competency, the ACGME, together with the American Board of Medical Specialties, published a suggested “toolbox” of assessment methods, including Chart Stimulated Recall; objective structured clinical examinations (OSCEs), and 360° Global Ratings [22]. Recent movement surrounding resident assessment focuses on achievement of particular milestones [23]. Previous studies describe the creation of an evaluation instrument in their resident-training program, and analyze the results as indicative of the effectiveness of the instrument and the training program [24–26].

In FM education-experienced environments, a gold-standard assessment would involve direct evaluation of a resident’s performance in patient care. A competency assessment would involve direct observation (e.g., Mini-CEX [27]) or review by a fully qualified family physician. Another option would be to examine board scores on a shelf examination each year (i.e., previously used test). Unfortunately, both procedures also require considerable infrastructure, including the availability of sufficient faculty time, shelf copies of a board examination (if one even exists) and a system to administer, score, and provide feedback to programs and residents. Unfortunately, both approaches are highly labor and cost intensive. A novel alternative would be a yearly resident self-competency evaluation. Using such an evaluation would provide a simple way of tracking individual development, and cumulatively, resident development in a program. This could thus be used to the extent newly developed FM residency training programs in FMEE countries are successfully teaching their residents the full breadth of care and providing reassurance that FM training program graduates with sufficient confidence in their knowledge and skills for FM practice.

In 2010 the University of Michigan Department of Family Medicine, along with three local hospitals, three mayors, and other supporters in Shizuoka Prefecture, Japan, received funding from the Prefectural Government to start a three-year FM training program unique in Japan for womb-to-tomb spectrum of care [28]. This research illustrates a longitudinal approach to self-competency assessments based on 5 years’ experience with the system in a new FM program in the FMEE country of Japan.

## Methods

The study utilized a longitudinal cross-sectional self-competency assessment survey conducted yearly during 2011–2015. The survey was distributed using an online survey system (Qualtrics) each spring, after the academic year ended, and repeated yearly over four years. The University of Michigan Institutional Review Board and Shizuoka FM Program (SFM), Shizuoka, Japan, approved this educational research.

The research was conducted with SFM program residents. Nine responses were collected in 2011, 12 in 2012, 13 in 2013, 12 in 2014, and 9 in 2015. Residents were asked to complete the self-assessment survey online in the spring, at the beginning of each residency year, and on completion of the program. At matriculation into the program, 12 residents started at postgraduate year (PGY) 3; 4 at PGY4; 1 at PGY5; 1 at PGY 6; and 2 at PGY9. This variation occurs as there is not a matching system beyond preliminary training programs [29]. All SFM residents participated voluntarily. To avoid confusion with their PGY, residents were categorized by their FM training year (FMY), e.g., FMY1 means first year of FM training.

In conjunction with U.S. and Japanese faculty, a pilot paper survey with 92 conditions was tested in the inaugural SFM class, 2010. When we initiated the study there was no “list of common” conditions in primary care in Japan. Hence, we adapted a list for piloting based on 87 common conditions reflecting womb-to-tomb conditions commonly seen in acute care, chronic care, minor procedures, and preventive care/health promotion. We used as a reference common conditions recommended for medical students to learn about during their 4 week, third year clinical rotation at the University of Michigan, along with 5 emergency procedure protocols used commonly in Japan. Over the ensuing years, some edits for clarification, and additions and deletions, were made (further details available on request). Since 2014, the final instrument used for analysis contained 142 items: 30 items regarding acute care conditions, 28 on chronic care, 8 on women’s health, 38 on procedures (including three physical examination skills of the male/female genitalia/breast), 21 on health promotion (including newborn/young child, adolescent, and adult male/female), 12 on geriatrics and home care, and 5 emergency procedures protocols [basic life support (BLS), advanced cardiac life support (ACLS), pediatric advanced life support (PALS), advanced life support in obstetrics (ALSO), Japan advanced trauma evaluation and care (JATEC)]. Participants ranked their confidence for each domain or skill using a 100-point visual analog scale; 0 represents “can’t do at all” and 100 indicates “can do satisfactorily/completely.” Most respondents completed the survey in 10–15 min.

Completed surveys were downloaded from Qualtrics and imported into Microsoft Excel for organization before further analysis with Statistical Computer Software R. Means for individual residents and classes were calculated for each item and group to determine an average score per year and for the class as a whole. Mean scores for each category were compared by year. To compare progress over the residency, mean scores were plotted by FM residency program year (FMY) using box-plots.

## Results

Twenty residents completed one or more of the 59 surveys received over the 5 years of instrument administration. Eleven respondents were women; 9 were men. By Japanese regulation, all residents must complete a two-year preliminary rotating residency before entering an advanced residency such as the SFM program. As not all residents took the survey all years, the number of surveys taken differed by FMY; 16 trainees completed the survey at baseline (matriculation), 17 trainees responded after completing FMY1, 11 after FMY2, and 11 after FMY3. Four trainees completed the survey only once, another five completed two surveys, three completed three surveys, and eight completed four surveys. For privacy reasons, data on age and previous residency training were not collected.

### Composite scores

**Fig. 1:**
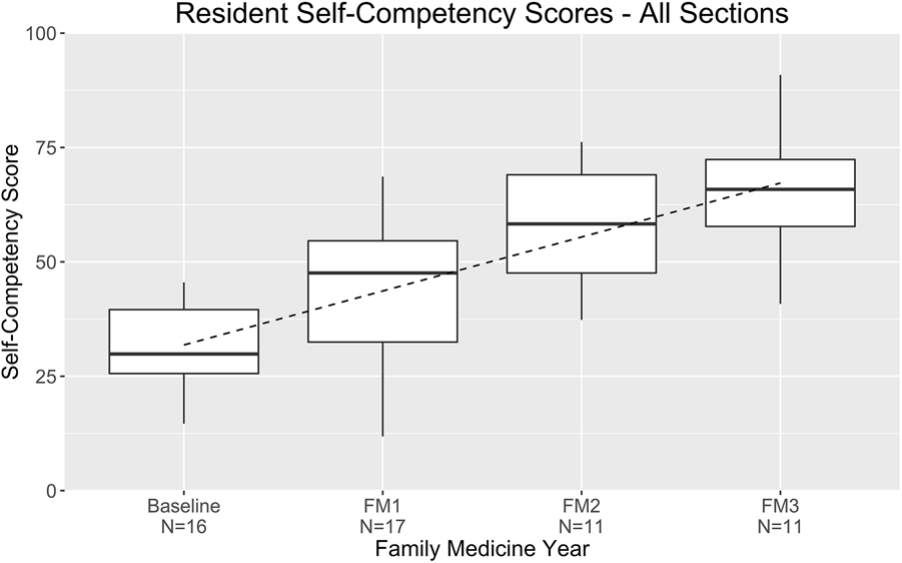
Resident self-competency assessments by year of training across the full spectrum of family medicine care

From the time of program entry to just after program completion three years later, mean self-competency evaluation scores increased almost linearly, from 31 to 65% (increase, 34% points) (Fig. 1). A moderate degree of distribution was observed at the PGY level on an individual basis.

### Acute conditions and emergency procedures

**Fig. 2:**
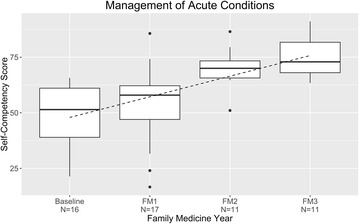
Management of acute conditions: resident self-competency assessments by year of training

**Fig. 3:**
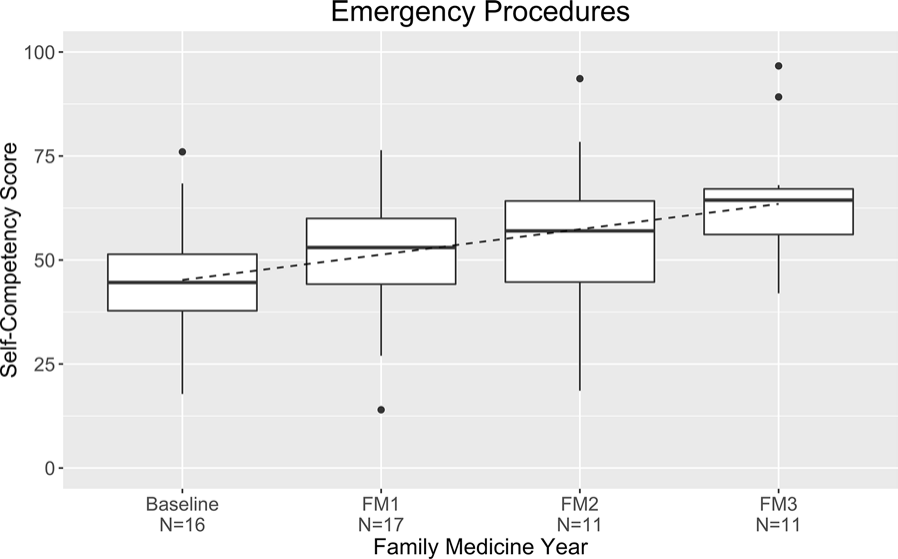
Management of emergency procedures: resident self-competency assessments by year of training

The mean score for acute conditions improved by 26% points over three years, from 49 to 75% (Fig. 2). The conditions evaluated were abdominal pain, asthma exacerbation, back pain, bronchitis, pneumonia, chest pain, dermatologic problem, dizziness, fever, fracture, gastroenteritis, musculoskeletal pain/strain/sprain, otitis media, pharyngitis/upper respiratory illness, sinusitis, urinary tract infection, headache, syncope, visual impairment, conjunctival injection, hearing impairment, hoarseness, nose bleed, palpitations, difficulty breathing, cough/phlegm, nausea/vomiting, heartburn, constipation, and melena/blood in stool. The emergency procedures surveyed—BLS, ACLS, PALS, ALSO, and JATEC—received a high self-rating at baseline, 46%, and peaked at 65% (Fig. 3).

### Chronic conditions

**Fig. 4:**
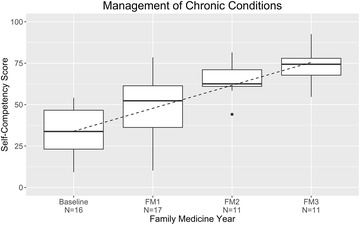
Management of chronic conditions: resident self-competency assessments by year of training

The mean score at baseline for chronic care management was 33% (Fig. 4). The score improved to 73% by the end of residency training (increase, 40% points); the largest increase occurred in the first year of residency. The chronic conditions assessed were acne, allergies, anemia, anxiety, arthritis, coronary artery/heart disease, cancer (any site), congestive heart failure, chronic obstructive pulmonary disease, dementia, depression, diabetes mellitus, fatigue, headaches, hyperlipidemia, hypertension, neurological disorder, obesity, smoking, substance abuse, pregnancy, warts, constipation, numbness/tingling, hematuria, dysuria, atopic dermatitis, and (chronic) benign prostatic hypertrophy.

### Chronic women’s health conditions

**Fig. 5:**
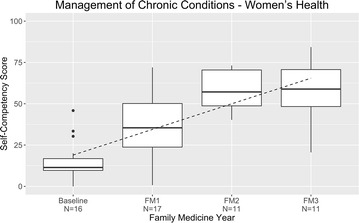
Management of chronic women’s health conditions: resident self-competency assessments by year of training

At baseline, the residents self-rated their competency at managing women’s health with a mean score of 16%, which climbed to 59% over their residency training (increase, 43% points) (Fig. 5). Skills involving amenorrhea/oligomenorrhea, contraception, dysfunctional uterine bleeding, sexually transmitted infections, urethritis, vaginitis, menopause/hormone-replacement therapy, and infertility were assessed.

### Outpatient procedures

**Fig. 6:**
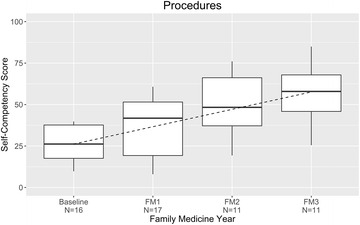
Management of core family medicine procedures: resident self-competency assessments by year of training

Mean self-assessments of competency regarding outpatient procedures rose only moderately, from 26% at baseline to 56% at residency graduation (Fig. 6). The survey included items on these procedures: dermatologic lesion biopsy, shave biopsy, dermatologic lesion removal, mole excision, cryotherapy, breast examination, casting/splinting, upper gastrointestinal endoscopy, abdominal ultrasonography, endometrial biopsy, male genitourinary examination, prostate examination, rectal examination, anoscopy, incision and drainage of abscess, joint aspiration/injection, pelvic examination, Papanicolaou smear, prenatal echocardiography, suturing, toenail removal, electrocardiogram interpretation, urinalysis, vaginal wet mount, gram stain, KOH test, otoscopy, ophthalmoscopy, gastrostomy management, central venous port management, urinary catheter management, echocardiography, gynecological ultrasonography, chest radiograph interpretation, abdominal radiograph interpretation, bone radiograph interpretation, central venous catheter insertion, and sebaceous cystectomy.

### Geriatrics, home care

**Fig. 7:**
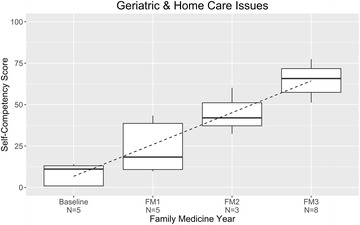
Management of geriatric/home care issues: resident self-competency assessments by year of training

Trainees’ self-assessment for geriatrics and management of home-care conditions, which began in 2013, started at a relatively low baseline, 8%, and rose rapidly, to roughly 65% (increase, 57% points) (Fig. 7). The survey items on geriatrics and management of home-care conditions assessed counseling for transitioning to home care, acute–home-care coordination, decubitus ulcer, urinary incontinence, fecal incontinence, home oxygen therapy, management of home intravenous drip including subcutaneous transfusion, fall-prevention management, home-ventilator management, multidisciplinary team coordination, terminal care, and end-of-life care.

### Preventive Services: Health Promotion, Screenings

**Fig. 8:**
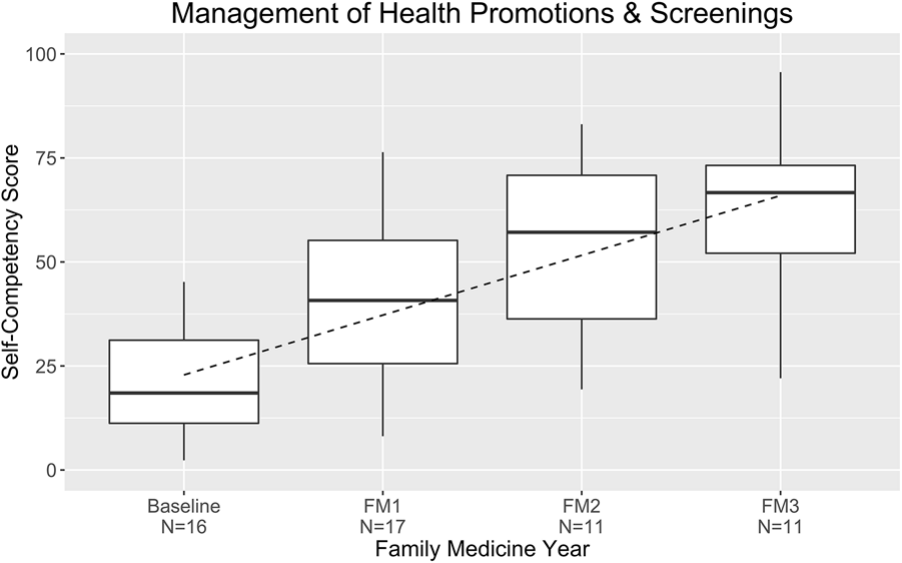
Management of health promotion and screening: resident self-competency assessments by year of training

Residents’ self-rated preventive services competency at baseline, a score of 21%, climbed steeply, to 63%, by residency graduation (increase, 42% points) (Fig. 8). The following services, by age-group, were assessed: newborn/infant examination—vaccination, child-rearing education, developmental milestones counseling; adolescents—sexual intercourse and contraception, alcohol consumption, illicit drug use, independence, suicidal ideation; adult—cardiovascular disease screening, hypertension screening, stress counseling, colorectal cancer screening, gastric cancer screening (a national priority in Japan), preventive aspirin administration, depression screening; female adult—cervical cancer screening, contraception, domestic violence screening, breast-cancer screening; male adult—smoking cessation, prostate-cancer screening.

## Discussion

These findings illustrate that if a FM residency training program provides sufficient training experiences, self-assessed competencies for a broad range of conditions and procedures can progressively improve over the course of training. The progressive improvements on the self-competency assessment findings suggesting successful skills in FM are corroborated by a passing performance by all residents who completed the program on the *actual official* national board examination conducted by the Japan Primary Care Association. This provides at least a minimal level of external validity of progressively improving self-competency assessments. For policy makers desiring FM womb-to-tomb training in three years, these data suggest FM residents can learn progressively on a year-to-year basis the content and skills necessary to feel confident in their ability to manage the acute care, chronic care, preventive care, and procedural care needed by patients in primary care.

### Implications for FM in Japan

Despite policy maker and educator lack of familiarity about FM residency programs’ ability to teach content outside of general internal medicine in Japan, these data provide compelling evidence that, in an appropriately structured program, residents can learn material and develop sufficient confidence in content areas, including women’s health, children’s health, and minor surgical specialties necessary for womb-to-tomb care. Of particular interest, the benefits of training in full-spectrum womb-to-tomb care were similar regardless of whether the learner had years of experience and practice in another non-generalist specialty or had just completed the basic clinical training through the 2-year mandatory rotating internship. Even residents at PGY5-9 at entry in the program initially scored low in areas of focus by family physicians, for example, preventive services, outpatient procedures, and women’s health.

These data are particularly timely as a requirement for women’s health care in *sougou shinryoui* training is under intense debate in Japan. There, many argue that women’s health care is the domain of obstetrician-gynecology providers exclusively. A plateau effect in progression occurred in the third year of residency. This probably occurred due to: limited opportunities to provide women’s health care in the outpatient FM clinics as FMY3s [30], a limited number of attending physicians trained in women’s health care, and patients’ being unaware that women’s health care services are provided in the FM clinics. Nonetheless, the evidence is clear that, given the opportunity, FM residents can develop the skills to provide competently women’s health care in Japan.

### Implications for FMEE countries

These self-competency assessments can be used potentially for residents to see their own progress and for programs to see the progress of residents, individually and across the board. Persistently low assessments by many residents might suggest a problem with rotations, internal or external. While other procedures regarded as gold standards for assessing resident progress and competency—for example, individualized assessments during patient care and monitoring of progress on shelf copies of mock examinations—are plausible, these approaches may be difficult to utilize in FMEE countries, where resources are limited. Regarding the performance of the instrument, conducting a concurrent validity assessment, by using faculty evaluations, was infeasible; however, the perception of faculty that residents were progressively improving in their ability to provide the breadth of care as expected with advancement in the program does suggest external validity of the instrument. The findings also are corroborated by a passing performance by all residents who completed the program on the national board examination conducted by the Japan Primary Care Association.

Although these surveys were conducted online, paper-based assessments would be possible. Additionally, considerable adjustments were necessary to make the categories relevant to Japan. Adding some procedures—for example, regarding the management of home-care conditions—was easy, while dropping others, e.g., vasectomy, was fairly obvious. Decisions regarding some conditions and procedures were difficult, however, because faculty were of different training backgrounds and faculty in the program come from diverse backgrounds; some instructors received training abroad, whereas others are self-styled family physicians who received traditional internal medicine training that excluded surgical care involving ophthalmology, otolaryngology, obstetrics/gynecology, urology, or general surgery. The biggest the self-competency assessment procedures challenge of using in another country would be developing the specific items in each category.

### Limitations

The mean scores reported represent a composite. Attrition or nonparticipation of certain residents over time precludes our inferring precise levels of competency, though making such pinpoint measurement was not the purpose of this study. Second, as rankings are self-assessments, no ideal score exists, particularly because of regional variations in services needed and provided. Third, there is no consensus in Japan and many FMEE countries about what FM residents should become competent to do. As the assessment using 142 items was informed by considerable U.S. experience, and adapted iteratively with input from Japanese FM colleagues, we believe it adequately represents most common problems and procedures in primary care in Japan. Home-care items were added to the survey to reflect national and prefectural priorities, though in future iterations programs may want to add items on complementary and alternative/integrative medicine, treatments commonly used in Japan, or other particular conditions or procedures emphasized. Fourth, breaking up the data was sometimes challenging, as some items related to women’s health were grouped with procedures and preventive health, while others are chronic conditions. Fifth, formal validity testing has not been conducted for this instrument, though face validity is self-evident.

## Conclusions

These data suggest that the SFM program provides a training experience that produces physicians confident to practice the breadth of care needed by family physicians. The findings further illustrate that appropriately structured FM training programs can produce graduates prepared to provide the full spectrum of FM care. A resident self-competency assessment provides a simple and practical way to conduct resident self-assessments of skills, to monitor this over time, to use the data to inform residency program improvement, and to demonstrate the breadth of family medicine training to policymakers, and other stakeholders.

## Abbreviations

FM: family medicine
FMEE: family medicine education emerging
JACME: Japan Accreditation Council for Medical Education
ACGME: Accreditation Council for Graduate Medical Education
PGY: postgraduate year
FMY: family medicine training year
BLS: basic life support
ACLS: advanced cardiac life support
PALS: pediatric advanced life support
ALSO: advanced life support obstetrics
JATEC: Japan Advanced Trauma Evaluation and Care
SFM: Shizuoka FM Program
